# Short-Term Surgical Outcomes and Definitive Diagnosis in Patients With Congenital Cardiac Defects: A Single-Center Analysis

**DOI:** 10.7759/cureus.74688

**Published:** 2024-11-28

**Authors:** Muhammad Aasim, Raheela Aziz, Atta ul Mohsin, Raheel Khan, Ayesha Zahid, Jibran Ikram

**Affiliations:** 1 Cardiac Surgery, Hayatabad Medical Complex Peshawar, Peshawar, PAK; 2 Cardiovascular Medicine, Hayatabad Medical Complex Peshawar, Peshawar, PAK

**Keywords:** cardiac surgery, congenital heart disease, septal defects, short term outcome, surgical outcomes

## Abstract

Background

With the rising number of children with congenital heart disease (CHD) reaching adulthood, surgical intervention has become a critical aspect of their long-term management. This study presents a six-year overview of early postoperative outcomes and mortality in CHD surgeries at a single center, underscoring advancements and challenges in treating this complex population.

Objective

As more children with CHD grow into adulthood, we are gaining critical insights from our extensive experience in performing cardiac surgery for this population. This report details our six-year experience at a single center, highlighting short-term outcomes and in-hospital mortality rates.

Methods

We collected data on all consecutive patients with CHD who underwent surgery between July 2018 and September 2024 in the Cardiac Surgery Department at Hayatabad Medical Complex, Peshawar, Pakistan. We evaluated early outcomes, including ventilation duration, length of intensive care unit (ICU) stay, total hospital stay, and mortality rates.

Results

A total of 250 procedures were performed on patients, with a mean age of 22.98 years (Range 1-78 years); 48.4% were male and 51.6% were female. Re-do procedures accounted for 2.4% of the total procedures. The most common procedures involved repair of septal defects (43.8%), right heart lesions (19.2%), and thoracic arterial and venous surgeries (16.8%). While the primary procedures involved less complex cases, they included 15.2% tetralogy of Fallot repairs, 4.0% aortic coarctation repairs, and 1.2% repairs for Ebstein’s disease. In-hospital mortality was recorded at 1.6%, with an overall survival rate of 98.4%.

Conclusion

Surgery in patients with CHD can be performed with low operative mortality and favorable clinical outcomes. Our findings demonstrate that, despite the complexities inherent to this population, most procedures yield successful results, contributing to a high overall survival rate. This underscores the importance of specialized surgical approaches and continuous management for CHD patients, emphasizing the potential for positive long-term outcomes in this growing demographic.

## Introduction

Congenital heart disease (CHD) is the most prevalent birth defect, affecting approximately one in every 100 live births [1]. In the United States, the incidence of CHD among premature infants, excluding isolated patent ductus arteriosus (PDA) and atrial septal defect (ASD), is 12.5 cases per 1,000 live births [2]. Despite improvements in detection and treatment, CHD still contributes to 3% of all infant deaths and 46% of fatalities resulting from congenital malformations [3]. The first successful surgical intervention for CHD, known as patent ductus arteriosus (PDA) ligation, was carried out by Dr. Robert Gross in 1938. Surgical procedures for other conditions were introduced over the following three to four decades. The survival outlook for patients with CHD has significantly improved in recent decades, primarily due to earlier diagnosis and advancements in surgical and percutaneous techniques tailored for this population [4]. Surgery performed at a younger age is primarily life-saving, whereas cardiac procedures performed later in life are typically elective and aim to alleviate symptoms or enhance prognosis. Today, about 90% of children born with CHD are reaching adulthood [5,6]. Due to significant advancements in pediatric cardiology, cardiac surgery, and intensive care, individuals with critical and complex CHD can now successfully reach adulthood. The surgical management of CHD in adults has experienced substantial growth recently, likely driven by a combination of improved diagnostic and treatment techniques, as well as increased patient awareness [7,8]. Some individuals with CHD may undergo primary surgery for defects that were either not treated in childhood or not detected early. Others might need reoperations after corrective surgeries or additional palliative procedures following an initial intervention [9]. Factors that affect outcomes are reduced myocardial function, decreased vascular compliance, increased arrhythmias, dysfunction of other organ systems, and age-associated comorbidities [10]. In this study, we aim to share our experiences and results over a six-year period at a single center, highlighting short-term outcomes and in-hospital mortality rates.

## Materials and methods

After institutional research and ethical board approval, we collected the data retrospectively on all patients who underwent CHD at a single tertiary care center, Hayatabad Medical Complex, Peshawar, Pakistan; between July 1, 2018 and September 30, 2024. Data regarding patient demographics, diagnoses, cardiac surgical procedures, and outcome parameters were gathered from the medical records. Collected data included ventricular function measured by ejection fraction (EF) by echocardiography, which was classified as either impaired (EF < 50%) or preserved (EF > 50%). A diagnosis of diabetes was considered present if there was a documented prior diagnosis. Pulmonary hypertension (PHT) was categorized based on systolic pulmonary artery pressure as: mild (less than 40 mmHg), moderate (between 40 mmHg to 60 mmHg), and severe (greater than 60 mmHg). The outcomes were predefined as the duration of mechanical ventilation, the lengths of stay in the intensive care unit (ICU) and the hospital, as well as in-hospital mortality. Statistical analysis was conducted using SPSS version 20 (IBM Corp., Armonk, NY, USA). Descriptive statistics for continuous variables are reported as mean and standard deviation and categorical variables are summarized as n (%).

Institutional Review Board approval was taken from Medical Teaching Institute-Hayatabad Medical Complex with the approval number 2303.

## Results

**Figure 1:**
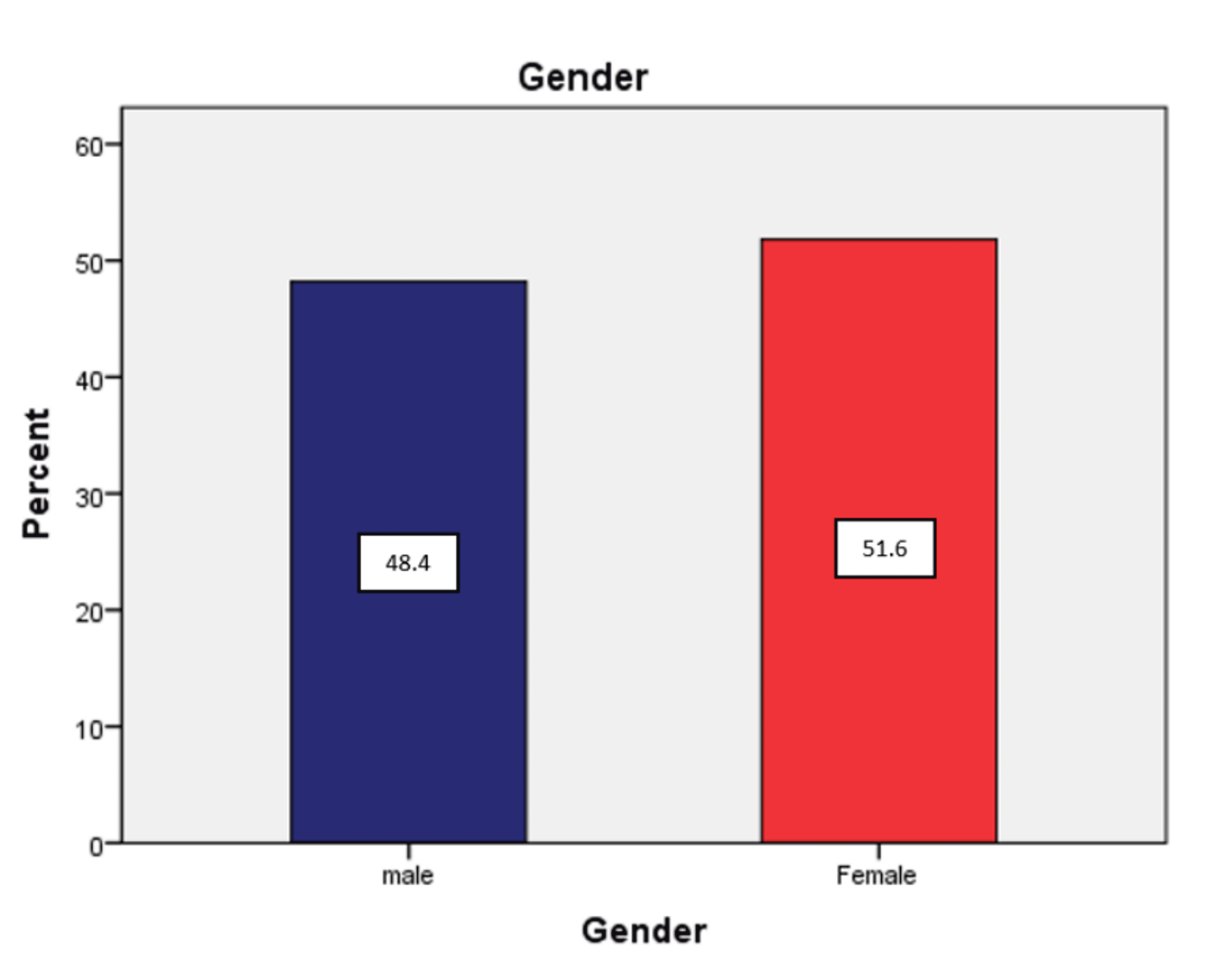
Gender distribution of patients undergoing congenital heart disease surgery (n=250)

During the study period, a total of 250 patients underwent initial surgery or a reoperation for CHD. Among the 250 patients, 121 were males (48.4%) and 129 females (51.6%) (Figure 1). 

The preoperative assessment of the patient cohort revealed significant insights into their cardiac health. Of the 250 patients evaluated, 234 (93.6%) exhibited preserved ventricular function, while 16 (6.4%) had impaired function. In terms of pulmonary hypertension, 153 (61.2%) were classified as having normal levels, whereas 52 (20.8%) experienced mild, 36 (14.4%) had moderate, and nine (3.6%) had severe hypertension. Additionally, comorbid conditions included diabetes in 22 (8.8%), active endocarditis in 16 (6.4%), and a prior cardiac surgery in six (2.4%). These findings highlight the diversity of preoperative conditions among the patients, which could impact surgical outcomes and influence postoperative care strategies (Table 1).

**Table 1:** Main characteristics of congenital heart disease (CHD) patients.

Variables	Number (%)
Sex	
Male	121 (48.4%)
Female	129 (51.6%)
Age (In years)	
Mean ± SD	22.98 ± 15.38
Range	1-78
Ventricular function:	
Preserved	234 (93.6%)
Impaired	16 (6.4%)
Pulmonary hypertension:	
Normal	153 (61.2%)
Mild	52 (20.8%)
Moderate	36 (14.4%)
Severe	9 (3.6%)
Diabetes	22 (8.8%)
Active Endocarditis	16 (6.4%)
Previous Cardiac surgery	6 (2.4%)

The average duration of ventilation was 7.33 ± 5.964 hours, length of stay in the ICU was 41.34 ± 8.291 hours and average hospital stay was 5.14 ± 0.662 days (Table 2).

**Table 2:** Perioperative parameters (early outcomes). ICU: intensive care unit, SD: standard deviation.

Variables	Mean ± SD
Ventilation (hours)	7.33 ± 5.964
ICU Stay (hours)	41.34 ± 8.291
Hospital Stay (days)	5.14 ± 0.662
In Hospital mortality (N)%-	4 (1.6%)

The definitive diagnoses of patients in our study encompassed a range of CHD. Among septal defects, 75 patients (30%) had secundum ASD, while six (2.4%) had sinus venosus ASD, and 21 (8.4%) presented with ventricular septal defects (VSD). Additionally, there was one (0.4%) case of partial ASD and two (0.8%) cases of complete atrioventricular septal defect.

Right heart lesions included 38 patients (15.2%) with Tetralogy of Fallot, three (1.2%) with Ebstein’s disease, and seven (2.8%) with pulmonary atresia associated with VSD. Thoracic anomalies featured 10 patients (4.0%) with aortic coarctation and 31 (12.4%) with PDA.

Electrophysiologic conditions included congenital heart block in 11 patients (4.4%), while pulmonary venous anomalies comprised one (0.4%) case of partial anomalous pulmonary venous connection and two (0.8%) cases of total anomalous pulmonary venous connection.

Lastly, left heart lesions included eight patients (3.2%) with subaortic stenosis. Additionally, several combinations of conditions were identified among the patients. Specifically, five patients (2.0%) had both ventricular septal defect and PDA, while three patients (1.2%) had ventricular septal defect with aortic stenosis.

Furthermore, four patients (1.6%) presented with ventricular septal defect and aortic regurgitation, and another five patients (2.0%) had ventricular septal defect alongside coronary artery disease. ASD was also noted in combination with various conditions: seven patients (2.8%) had severe tricuspid regurgitation, four (1.6%) had mitral regurgitation, and three (1.2%) had severe mitral stenosis.

**Figure 2:**
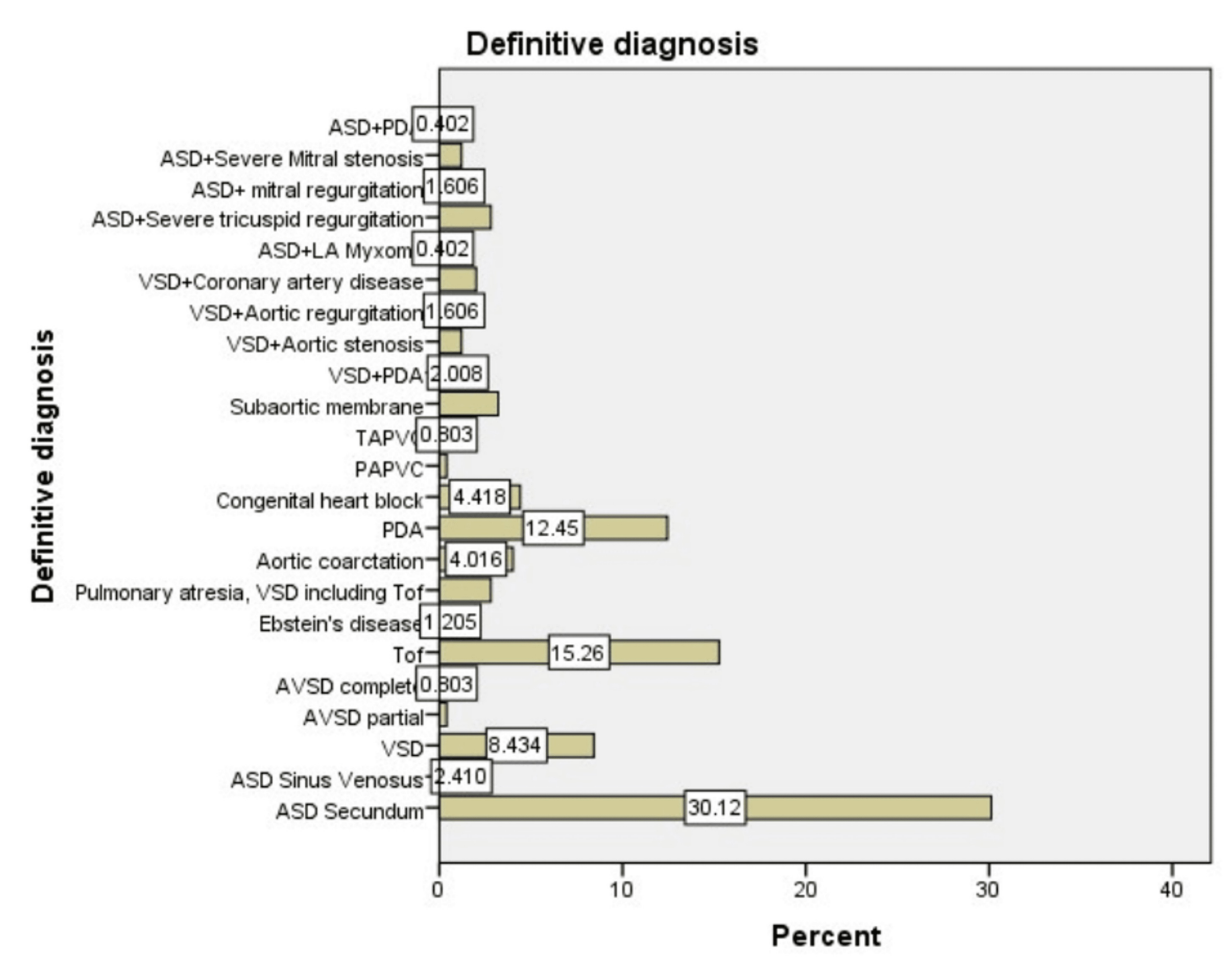
Definitive diagnosis ASD: Atrial septal defect, LA: Left atrium, VSD: Ventricular septal defect, PDA: Patent ductus arteriosus, TAPV: Total Anomalous pulmonary venous connection, PAPVC: Partial anomalous pulmonary venous connection, TOF: Tetralogy of Fallot

Additionally, one patient (0.4%) presented with ASD and left atrial myxoma, while another patient (0.4%) had ASD combined with PDA. These findings illustrate the complexity of CHD in the cohort (Figure 2, Table 3).

**Table 3:** Primary anatomic diagnosis PA: Persistent arteriosus, LA: Left atrium, VSD: Ventricular septal defect, PDA: Patent ductus arteriosus, TOF: Tetralogy of Fallot

Definitive Diagnosis	Number (%)
Septal defects	
- Atrial septal defect, secundum	75 (30%)
- Atrial septal defect, sinus venosus	6 (2.4%)
- Ventricular septal defect	21 (8.4%)
- Atrio-ventricular septal defect, partial	1 (0.4%)
- Atrio-ventricular septal defect, complete	2 (0.8%)
Right heart lesions	
- Tetralogy of Fallot	38 (15.2%)
- Ebstein’s disease	3 (1.2%)
- Pulmonary atresia, VSD including TOF/PA	7 (2.8%)
Thoracic arteries & veins	
- Aortic coarctation	10 (4.0%)
- Coronary artery anomaly, origin	1 (0.4%)
- Patent ductus arteriosus	31 (12.4%)
Electrophysiologic	
- Arrhythmia, heart block, congenital	11 (4.4%)
Pulmonary venous anomalies	
- Partial anomalous pulmonary venous connection	1 (0.4%)
- Total Anomalous pulmonary venous connection	2 (0.8%)
Left heart lesion	
- Subaortic membrane (subaortic stenosis)	8 (3.2%)
Others	
- Ventricular septal defect + PDA	5 (2.0%)
- Ventricular septal defect + Aortic stenosis	3 (1.2%)
- Ventricular septal defect + Aortic regurgitation	4 (1.6%)
- Ventricular septal defect + Coronary artery disease	5 (2.0%)
- Atrial septal defect + Severe Tricuspid regurgitation	7 (2.8%)
- Atrial septal defect + Mitral regurgitation	4 (1.6%)
- Atrial septal defect + Severe Mitral stenosis	3 (1.2%)
- Atrial septal defect + LA myxoma	1 (0.4%)
- Atrial septal defect + PDA	1 (0.4%)

Septal defect repairs included 75 cases (30%) of secundum ASD repair, six cases (2.4%) of sinus venosus ASD repair, and 21 cases (8.4%) of VSD repair. Additionally, there were one (0.4%) partial and two (0.8%) complete atrioventricular septal defect repairs. Right heart lesions were addressed with three (1.2%) Ebstein’s repairs and 38 (15.2%) total corrections for tetralogy of Fallot (TOF). Left heart lesions were treated with eight (3.2%) subaortic membrane resections. Electrophysiologic procedures included 11 patients (4.4%) receiving pacemakers. Thoracic interventions consisted of 10 cases (4.0%) of end-to-end coarctation repair, 31 (12.4%) PDA closures, and one (0.4%) coronary artery bypass for anomalous origin. Palliative care involved seven (2.8%) central shunt procedures. Lastly, pulmonary venous anomalies were treated with one (0.4%) repair of partial anomalous pulmonary venous connection and two (0.8%) repairs of total anomalous pulmonary venous connection. These procedures reflect the diverse surgical management in our patient population. Surgical procedures performed on patients with congenital heart defects are outlined in Table 4.

**Table 4:** Procedures performed on patients with congenital heart defects. PDA: Patent ductus arteriosus

Main Surgeries	Number (%) (n=250)
Septal Defects	
- Atrial septal defect Secundum repair	75 (30%)
- Atrial septal defect Sinus venosus repair	6 (2.4%)
- Ventricular septal defect repair	21 (8.4%)
- Partial atrioventricular septal defect repair	1 (0.4%)
- Complete atrioventricular septal defect repair	2 (0.8%)
Right Heart Lesions	
- Ebstein’s repair	3 (1.2%)
- Total correction for Tetralogy of Fallot	38 (15.2%)
Electrophysiologic	
- Pacemaker procedure	11 (4.4%)
Thoracic Arteries & Veins	
- Coarctation repair, end-to-end	10 (4.0%)
- PDA closure	31 (12.4%)
- Coronary artery bypass for anomalous origin of coronary artery	1 (0.4%)
Palliative Procedures	
- Shunt, central	7 (2.8%)
Pulmonary Venous Anomalies	
- Partial anomalous pulmonary venous connection repair	1 (0.4%)
- Total anomalous pulmonary venous connection repair	2 (0.8%)
Left Heart Lesions	
- Subaortic membrane resection	8 (3.2%)

A total of 33 surgical interventions (13.2%) involved various combinations of procedures. A total of five patients (2.0%) underwent VSD closure along with PDA ligation, while three patients (1.2%) received both VSD closure and aortic valve replacement. Additionally, four patients (1.6%) had VSD closure combined with aortic valve repair, and another five patients (2.0%) underwent VSD closure with coronary artery bypass grafting. For ASD procedures, seven patients (2.8%) had closure combined with tricuspid valve repair, four patients (1.6%) had closure with mitral valve repair, and three patients (1.2%) underwent closure with mitral valve replacement. Furthermore, one patient (0.4%) had ASD closure along with left atrial myxoma resection, and another patient (0.4%) received closure combined with PDA ligation (Table 5).

**Table 5:** Concomitant procedures. ASD: Atrial septal defect, LA: Left atrium, VSD: Ventricular septal defect, PDA: Patent ductus arteriosus

Surgeries	Number (%)
VSD closure + PDA ligation	5 (2.0%)
VSD closure + Aortic valve replacement	3 (1.2%)
VSD closure + Aortic valve repair	4 (1.6%)
VSD closure + Coronary artery bypass grafting	5(2.0%)
ASD closure + Tricuspid valve repair	7(2.8%)
ASD closure + Mitral valve repair	4 (1.6%)
ASD closure + Mitral valve replacement	3 (1.2%)
ASD closure + LA myxoma resection	1 (0.4%)
ASD closure + Patent ductus arteriosus ligation	1 (0.4%)

These combinations reflect the complexity of surgical management in this patient population. In our study, six (2.4%) patients underwent surgery as a redo for congenital heart defects that had been primarily addressed during childhood (Table 1).

## Discussion

Outcomes following CHD surgeries have significantly improved over recent decades due to advances in surgical techniques, anesthesia, and perioperative care. Our six years of experience, encompassing over 250 procedures for patients with CHD in a specialized adult cardiac surgery department, provides a valuable foundation for reflecting on and strategizing healthcare for this unique patient population in Pakistan. Advancements in diagnostic tools and enhanced medical management have enabled most newborns with CHDs to reach adulthood. However, only those with relatively simple conditions, such as isolated VSD, PDA without pulmonary hypertension, or ASD, are typically cured through primary cardiac surgery [11]. Consequently, the short- and long-term outcomes of adults with CHD have emerged as a crucial area of growing clinical interest. In the present era, patient presentations, in Pakistan, show considerable variation. Some individuals undergo primary surgery for defects that were not addressed during childhood, whether due to lack of recognition or missed follow-up. Meanwhile, others require redo surgeries to address complications arising from previous palliative procedures. In our study, the redo procedures primarily originated from other cities in Pakistan or neighboring Afghanistan, with the exception of one case of total correction for TOF that had previously undergone a palliative procedure at our center. A palliative procedure was proposed for the patients with complex CHD, aiming to temporarily enhance their clinical condition. While these palliative operations such as central shunts effectively reduced cyanosis with a relatively low hospital mortality rate of 0% in our study. Overall, we recorded six redo cases, in-hospital mortality rate was 4%, which is comparable to findings from studies conducted at tertiary care centers specializing in adult congenital heart surgery, such as the one by Niwa et al. [12]. When we compare our results to those of larger multicenter studies, early mortality rates are notably low, falling between 1.7% and 3.1%. Our study indicates an overall survival rate of 98.4%, which is favorable for corrective procedures. In our study, the average length of stay and ventilation duration were notably short, and well comparable with findings from other research [11,13]. None of the patients in the study required chest re-exploration due to bleeding. We conducted this study to assess short-term outcomes in patients with CHD. Our findings demonstrate favorable mortality and survival rates that are comparable to those from various centers worldwide. Given the increasing diversity within this patient population, it is essential for researchers and clinical teams to intensify their efforts to address patient needs and enhance their quality of life.

This study has some limitations, as a single-center analysis, the findings may not be generalizable to all CHD centers, especially those with different patient demographics or resource limitations. Future prospective studies across multiple centers are recommended to validate these findings and explore long-term outcomes.

## Conclusions

Our findings on surgery for CHD suggest that low mortality and minimal serious morbidity can be achieved at a tertiary referral center. However, further research is needed to explore key areas such as decision-making for this patient population, the optimal timing for interventions, and the development of more accurate predictive models, especially considering the diverse range of anatomical diagnoses and surgical procedures involved. Despite these positive outcomes, continued efforts are necessary to extend access to CHD care and resources, especially in underserved areas, to ensure that all CHD patients, regardless of geography, can benefit from these advancements. Future multi-center, long-term studies are warranted to further elucidate factors impacting morbidity and survival in adult CHD populations, especially as they age and acquire additional comorbidities.
